# Incidence, Demographics, and Outcomes of Penetrating Trauma in Sweden During the Past Decade

**DOI:** 10.3389/fneur.2021.730405

**Published:** 2021-11-15

**Authors:** Mattias Günther, Martin Dahlberg, Amir Rostami, Ali Azadali, Ulf P. Arborelius, Fredrik Linder, Elham Rostami

**Affiliations:** ^1^Department of Neuroscience, Karolinska Institutet, Stockholm, Sweden; ^2^Department of Clinical Science and Education Södersjukhuset, Karolinska Institutet, Stockholm, Sweden; ^3^Department of Social Work and Criminology, University of Gävle, Gävle, Sweden; ^4^Institute for Future Studies, Stockholm, Sweden; ^5^Department of Surgical Sciences, Uppsala University, Uppsala, Sweden; ^6^Department of Neuroscience, Section for Neurosurgery, Uppsala University, Uppsala, Sweden

**Keywords:** penetrating head trauma, penetrating trauma, epidemiology, head and neck injury, gunshot trauma, stab wound

## Abstract

Trauma injury is the sixth leading cause of death worldwide, and interpersonal violence is one of the major contributors in particular regarding injuries to the head and neck. The incidence, demographics, and outcomes of penetrating trauma reaching hospitals in Sweden are not known. We report the largest, nationwide epidemiological study of penetrating injuries in Sweden, using the Swedish Trauma Registry (SweTrau). A multi-center retrospective descriptive study of 4,776 patients was conducted with penetrating injuries in Sweden, between 2012 and 2018. Due to the increase in coverage of the SweTrau registry during the same period, we chose to analyze the average number of cases for the time intervals 2013–2015 and 2016–2018 and compare those trends to the reports of the Swedish National Council for Crime Prevention (Brå) as well. A total of 663 patients had Injury Severity Score (ISS) ≥ 15 at admission and were included in the study. Three hundred and sixty-eight (55.5%) were stab wounds (SW), 245 (37.0%) gunshot wounds (GSW), and 50 (7.5%) other traumas. A majority of the cases involved injuries to the head, neck, and face. SW increased from 145 during 2013–2015 to 184 during the second period of 2016–2018. The increase was greater for GSW from 92 to 141 during the same respective periods. This trend of increase over time was also seen in head, neck, and face injuries. The 30-day mortality was unaffected (48–47%) in GSW and trended toward lower in SW (24–21%) when comparing 2013–2015 with 2016–2018. Patients with head trauma had 45% mortality compared to 18% for non-head trauma patients. Head trauma also resulted in worse outcomes, only 13% had Glasgow outcome score (GOS) 5 compared to 27% in non-head trauma. The increasing number of cases of both SW and GSW corresponded well with reports from Brå although further studies also are needed to address deaths outside of hospitals and not registered at the SweTrau. The majority of cases had injuries to the head, neck, and face and were associated with higher mortality and poor outcomes. Further studies are needed to understand the contributing factors to these worse outcomes in Sweden and whether more targeted trauma care of these patients can improve outcomes.

## Introduction

Traumatic injury is the overall leading cause of death under 45 years of age worldwide resulting in an annual worldwide death toll of more than 5.8 million people. The trauma population has a low mean age, and more than two-thirds of patients are completely healthy before the traumatic event (1–3). The predominant cause of death is due to head and neck injuries followed by bleeding as the second most frequent cause (4, 5). Although the mechanism of injury may vary by region, interpersonal violence is one of the top three causes of traumatic injuries (6). The presence of illegal weapons and gun violence is increasing challenges in European countries (7), and there is a concern that injuries and deaths caused by violent actions are increasing, but only a few European epidemiological studies of civilian penetrating injuries have been published (8, 9). The rate of firearm-related homicide victimization among men aged 15–29 years is higher in Sweden compared to other Western European countries (10). The Swedish National Council for Crime Prevention (Brå) reports that the number of cases of lethal violence from firearms has more than doubled, from 17 cases in 2011 to 45 total cases in 2019 (11). Furthermore, Sweden has a larger proportion of homicide from firearms (35%) compared to the overall European level (13%) (10), and one trauma center in Sweden reported a significant increase in firearm injuries between 2005 and 2016 (12). However, the national incidence and demographics of penetrating trauma reaching trauma care in Sweden are not known, and the healthcare management and outcomes of these penetrating injuries have not been reported in the scientific literature. The aim of this study was to investigate trends and characteristics in the distribution of penetrating trauma in Sweden during 2012–2018 and in particular penetrating injuries to the head and neck. Data were derived from Swedish Trauma Registry (SweTrau), the national trauma registry in Sweden. The primary outcome was the incidence of penetrating injuries during the study period, and secondary outcomes were 30-day mortality, injury locations, emergency procedures, age/sex demographics, and day/time distributions.

## Methods

### Study Population

A multi-center retrospective study of all patients registered in SweTrau, with penetrating injuries in Sweden, between 2012 and 2018 was performed. Patients of all ages admitted to trauma centers were included. The population in Sweden was 9,555,893 in 2012 and 10,230,185 in 2018. The study was approved by the Swedish Ethical Review Authority (no 2019-02842) and by the SweTrau steering group. The requirement of informed consent was waived.

### The SweTrau and Participating Hospitals

The SweTrau is a national trauma registry in Sweden established in 2011 based on “the revised Utstein Trauma Template for Uniform Reporting of Data following Major Trauma, 2009” (13). The registry includes all patients where a full or limited trauma alert has been activated and all trauma patients with an Injury Severity Score (ISS) > 15. As of 2019, Sweden has 50 hospitals with trauma capabilities (acute care surgery, anesthesia, and radiology available around the clock), of which 46 (92%) report to SweTrau and 43 (86%) report actively (14). SweTrau publishes annual reports, such as the coverage and reporting degree of the registry (14). The coverage was estimated by SweTrau, from the number of patients with a trauma diagnosis and intensive care needs in SweTrau divided by the number of care sessions in the Swedish Intensive Care Registry (SIR) (www.icuregswe.org) with admission diagnosis “Trauma” and injury diagnosis SA01-TA04 and TA09-TA13. This was applied as one tool for evaluating the reliability of the data.

### Transferred Patients and Multiple Occurrences

A few patients were recorded for trauma with ISS > 15 on more than one occasion (three patients, maximum of two admissions) as identified by more than one date of trauma. Some patients occurred multiple times in the register due to data entry made at different hospitals (transferred patients). For such patients, we collected all injuries observed at the different sites and counted hospital days from admission at the first hospital to discharge from the last hospital. Ventilator time, for a particular patient, was counted at the site with most patient days on the ventilator. The maximum ISS score attributed was used. For other variables, the first data entry for each respective variable was used. Duplicated data for a subgroup of patients were pruned, keeping the latest version of the data.

### Comparative Trend/Register Analyses

To investigate how well the data on penetrating injuries registered with SweTrau corresponded to other registries in Sweden, we used data from Brå to compare trends of penetrating trauma (SweTrau) with confirmed cases of lethal violence, with and without firearms. The Brå is an agency under the auspices of the Swedish Ministry of Justice and is responsible for the official Swedish criminal statistics (15).

### Statistical Analyses

Data preparation was done with R (v 3.5.1). Descriptive statistics were used for patient characteristics and outcomes. The data presented constitute the entire population registered in SweTrau. For this reason, no test of statistical inference was used, as probability tests aim to assess a sample size in relation to a population.

## Results

The study analyzed 4,776 patients treated for penetrating trauma between 2012 and 2018. The three largest urban areas in Sweden, Stockholm, Gothenburg, and Malmö, received a total of 2,446 (51.2%) of the patients as the primary admitting hospital, i.e., Karolinska University Hospital, Stockholm 1,343 (28.1%), Sahlgrenska University Hospital, Gothenburg 602 (12.6%), and Skåne University Hospital, Malmö 501 (10.5%). Some 4,113 (86%) had an ISS <15 and 663 (14%) had an ISS ≥15. To assess major and severe trauma caused by penetrating injuries, we only included patients with ISS ≥ 15 for the following analyses. To facilitate the comparison of trends, we analyzed two time periods, 2013–2015 and 2016–2018.

The characteristics of the penetrating trauma are shown in Table 1. Only one injury mechanism was documented for each patient as the primary injury. Three hundred and sixty-eight (55.5%) were stab wounds (SW), 245 (37.0%) gunshot wounds (GSW), and 50 (7.5%) other penetrating injury mechanisms. This heterogeneous group of other injuries (Table 2) was excluded from further analysis. In this study, we, thus, focus on the two largest injury mechanisms, SW and GSW, comprising 613 patients (92.4%) of all penetrating trauma the majority of cases were assaults (Table 2). The majority of GSW had injuries to the head, neck, and face: during 2013–2015 (59%) and 2016–2018 (50%). For SW, the dominating region was the thorax followed by injuries to the head, neck, and face, with the latter being reported as injured in 48 and 40% in the 2013–2015 and 2016–2018 time periods, respectively. A total of 166 (25%) patients had one injury and 497 (75%) patients had injuries to multiple regions.

**Table 1:** Characteristics of the patients.

			**GSW**		**SW**	
		**All (2012–2018)**	**2013–2015**	**2016–2018**	**2013–2015**	**2016–2018**
Age (years)		30 [22, 45)	28 [22, 43]	25 [22, 37]	31 [23, 45]	30 [22, 43]
Total		663	92	141	145	184
Sex	Female	60	2	3	16	22
	Male	603	90	138	129	162
ISS	15–24	324	29	48	83	120
	25–49	255	50	63	44	51
	50–75	84	13	30	18	13
ASA	1	451	64	97	111	115
	2	117	13	21	22	43
	3	39	4	9	4	16
	Unknown	56	11	14	8	10
Requiring ventilator	Days	2 (1, 4)	2 (1, 6)	2 (1, 6)	1 (1. 4)	1 (1, 3)
	Number	302	48	70	57	77
Not requiring ventilator	Number	361	44	71	88	107
Hospital stay (days)		5 (2, 11)	4.5 (2, 13)	4 (1, 18)	6 (3, 10)	6 (2, 9)
Region (fraction)	Head	198 (29.9%)	42 (45.7%)	49 (34.8%)	37 (25.5%)	35 (19.0%)
	Face	159 (24%)	30 (32.6%)	37 (26.2%)	31 (21.4%)	38 (20.7%)
	Neck	97 (14.6%)	7 (7.6%)	16 (11.3%)	29 (20.0%)	35 (19.0%)
	Thorax	417 (62.9%)	45 (48.9%)	73 (51.8%)	109 (75.2%)	134 (72.8%)
	Abdomen	277 (41.8%)	40 (43.5%)	68 (48.2%)	58 (40.0%)	77 (41.8%)
	Spine	93 (14.0%)	14 (15.2%)	26 (18.4%)	13 (9.0%)	17 (9.2%)
	Upper extremity	251 (37.9%)	34 (37.0%)	54 (38.3%)	56 (38.6%)	73 (39.7%)
	Lower extremity	207 (31.2%)	28 (30.4%)	76 (53.9%)	31 (21.4%)	50 (27.2%)
	External	124 (18.7%)	9 (9.8%)	8 (5.7%)	37 (25.5%)	35 (19.0%)
Systolic BP measured (mmHg)	120 (94, 140)	125 (98.5, 150) 114.5	(89.5, 142.5)	117 (90, 138)	120 (97, 140)
Cardiac arrest on admission		51	8	15	15	11
Systolic BP (palpable at carotid)		80	14	28	9	18
1 (only palp at carotid)		9	2	2	0	3
2 (palpable femoral)		4	0	2	0	1
3 (weak radial)		6	1	2	1	1
4 (radial)		13	1	3	3	4
Unknown		22	4	4	7	4
GCS	≤ 8	118	28	37	17	19
	9–12	24	2	4	6	8
	≥13	351	35	55	88	113
Intubated		132	26	34	24	31
Unknown		38	1	11	10	13
30 day mortality dead		207	43	66	34	38
Alive		447	46	73	110	144
Unknown		9	3	2	1	2
Age brackets	0–14	10	4	0	2	3
	15–29	313	45	85	69	86
	30–44	169	20	31	34	55
	>45	166	22	25	40	40
Unknown		5	3	0	0	0

*The numbers are given as median [IQR], for dichotomous values it is given as mean*.

*GSW, gunshot wounds; SW, stab wounds*.

**Table 2:** Intent and injury mechanism for all and head trauma patients.

		**2012–2018**	**2013–2015**	**2016–2018**
Intent (all patients) *n* = 663	Accidental	67 (10.1%)	29 (11.2%)	36 (10.3%)
	Self-inflicted	94 (14.2%)	40 (15.4%)	45 (12.9%)
	Assault	480 (72.4%)	187 (71.9%)	251 (71.9%)
	Other	4 (0.6%)	1 (0.4%)	3 (0.9%)
	Unknown	18 (2.7%)	3 (1.2%)	14 (4%)
Intent (head trauma) *n* = 315	Accidental	33 (10.5%)	16 (11.7%)	16 (10%)
	Self-inflicted	61 (19.4%)	30 (21.9%)	26 (16.3%)
	Assault	207 (65.7%)	89 (65.0%)	106 (66.3%)
	Other	0 (0%)	0 (0%)	0 (0%)
	Unknown	14 (4.4%)	2 (1.5%)	12 (7.5%)
Mechanism of injury	MVA	9		
	MC	4		
	Bicycle	1		
	Pedestrian	1		
	Other vehicle	1		
	GSW	245		
	Stab	368		
	Blunt	7		
	Same level	6		
	Fall from height	7		
	Explosion	7		
	Other	7		
	Unknown	0		

*GSW, gunshot wounds*.

The procedures performed in the hospital are shown in Table 3. The most common procedures were wound management in 199 (30.0%) cases, insertion of a chest tube in 179 (27.0%) cases, laparotomy in 172 (25.9%) cases, and thoracotomy in 134 (20.2%) cases. Only 6.3% of patients underwent cranial procedures and 3.8% had intracranial pressure (ICP) monitoring.

**Table 3:** Emergency procedures on admission.

**Procedures**	**All cases (*n* = 663)**	**GSW (*n* = 245)**	**SW (*n* = 368)**
Wound management	199	64	121
Chest tube	179	64	109
Laparotomy	172	78	89
Thoracotomy	134	53	77
Orthopedic	86	40	32
Vascular	65	29	34
Cranial	42	22	15
ENT/MaxFac/Eye	31	13	15
Endoscopy	30	15	14
ICP monitoring	25	19	5
Tracheotomy	25	18	6
Interventional radiology	22	5	15
Fasciotomy	18	13	3
Laparoscopy	16	2	13
Spine	14	6	6
Pericardial drain	7	3	4
Urological	7	3	4
Thoracoscopy	2	1	1
ECMO	1	1	0

**Figure 1:**
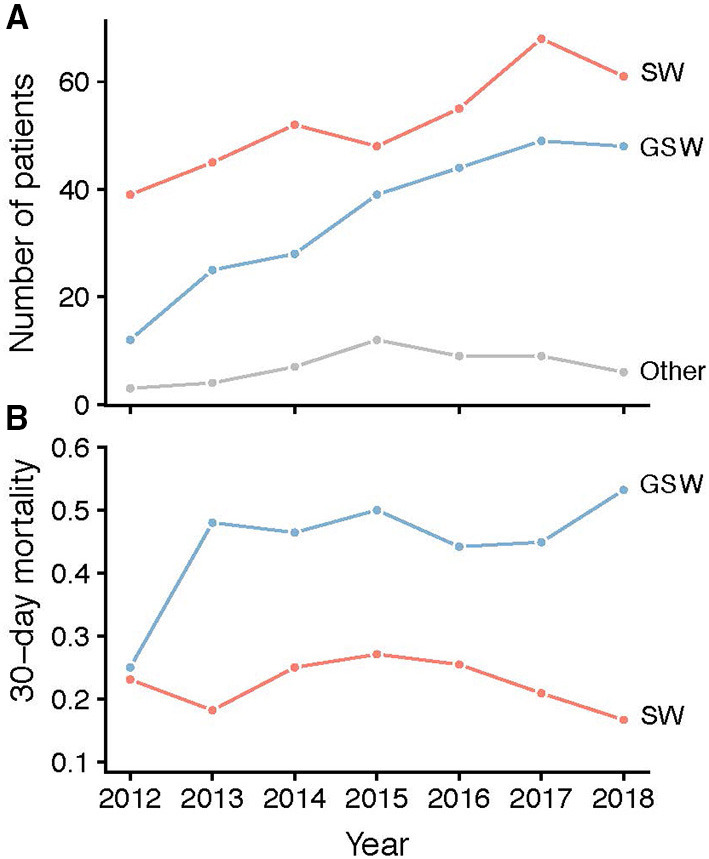
**(A)** Time development of the number of patients per year with stab wound (red), GSW (blue), or other (gray) trauma mechanisms. **(B)** Time development of 30-day mortality (fraction) in the stab wound (red) and GSW (blue) subgroups. GSW, gunshot wounds.

**Figure 2:**
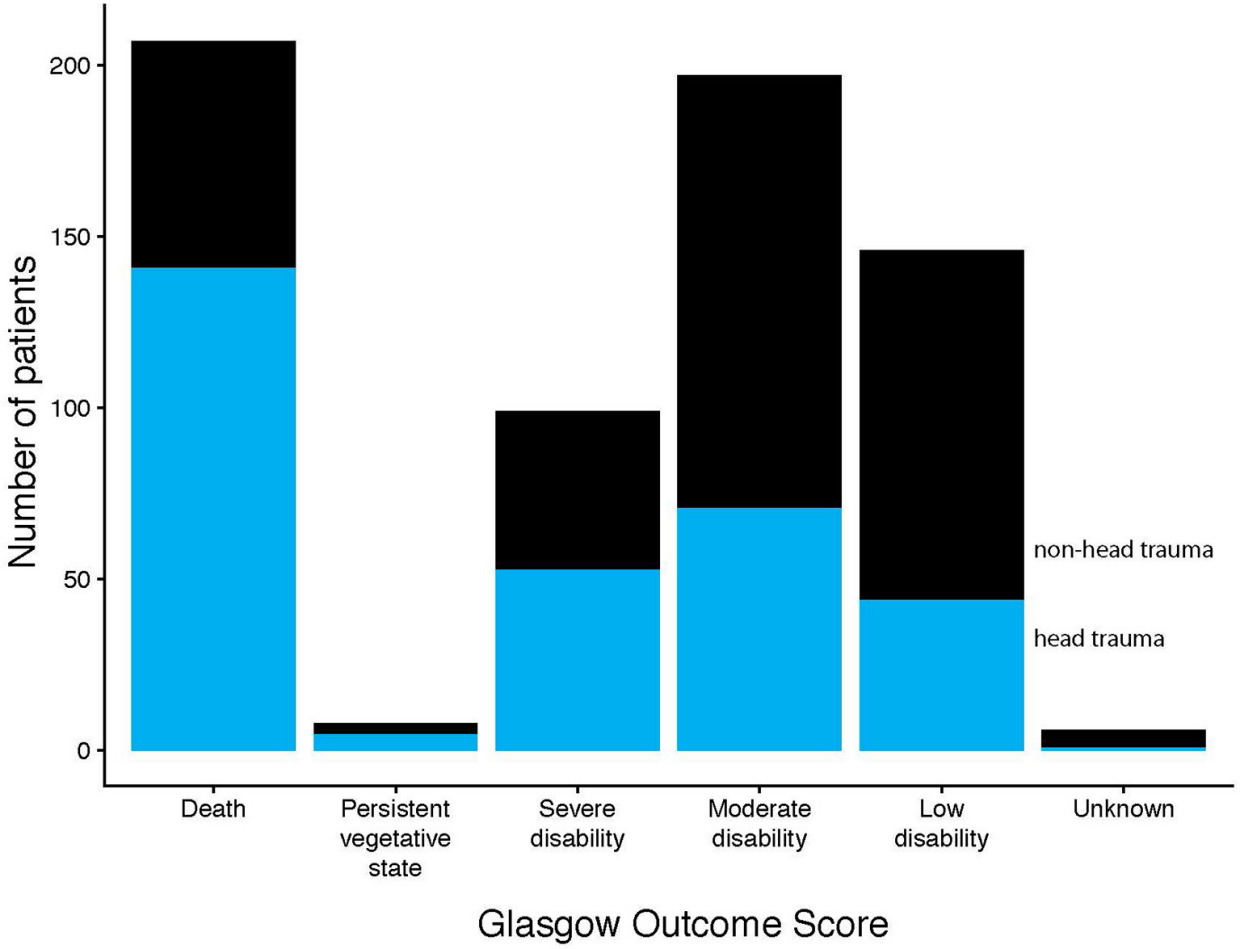
Illustrates the number of patients in each GOS category at discharge with and without head trauma. GOS 1 = dead, 2 = persistent vegetative state, 3 = severe disability, 4 = moderate disability, and 5 = low disability.

In the registry, SW was increased by 64% from 39 in 2012 to 61 in 2018, and during the same period, GSW was increased by 300% from 12 in 2012 to 48 in 2018 (Figure 1A). There was also an increase between time periods 2013–2015 (*n* = 92) and 2016–2018 (*n* = 141). However, the 30-day mortality was unchanged (48 and 47%, respectively) in GSW and was slightly lower in SW (24 and 21%, respectively) between the time periods 2013–2015 and 2016–2018 (Figure 1B). In the group with injuries to the head, face, and neck, the proportion of patients who had died at 30 days was higher in GSW patients (34/54, 63% in 2013–2015 and 49/71, 69% in 2016–2018) than in SW patients (19/69, 28% in 2013–2015, and 22/74, 30% in 2016–2018). In addition, patients with head trauma had 45% mortality (*n* = 134). Only 13% of these patients had favorable outcomes according to GOS at discharge (Figure 2) compared to non-head trauma patients where 27% had GOS 5.

**Figure 3:**
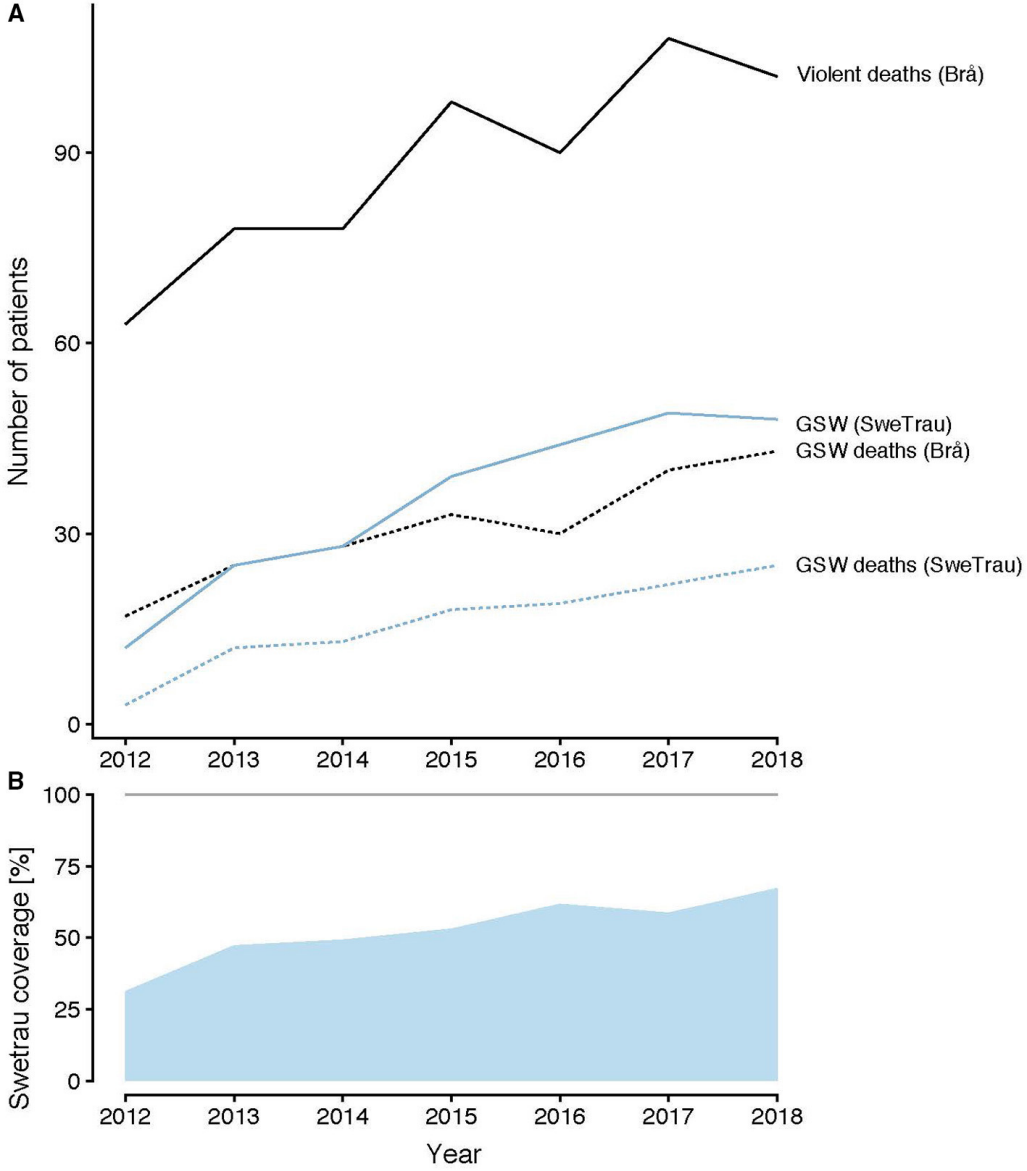
**(A)** Cases of violent deaths (black) and GSW deaths (black dotted), statistics from Brå. Cases of GSW from SweTrau (blue) and GSW deaths (blue dotted) from SweTrau. **(B)** SweTrau coverage [the number of patients with a trauma diagnosis and intensive care needs in SweTrau divided by the number of care sessions in the Swedish Intensive Care Registry (SIR)]. GSW, gunshot wounds.

The number of cases in SweTrau was compared with data from Brå and displayed similar trends (Figure 3A). The SweTrau coverage (the number of patients with a trauma diagnosis and intensive care needs in SweTrau divided by the number of care sessions in the SIR) is displayed in Figure 3B and shows an increasing coverage during the study period, from 31.3% in 2012 to 74.7% in 2018.

However, as SweTrau states in its reports (https://rcsyd.se/swetrau/om-swetrau/arsrapporter), the larger cities with the highest number of traumas, such as Stockholm, Uppsala-Örebro, and Malmö, have high coverage of around 90% early on. SweTrau also states in its report a yearly increase of both actual numbers and the percentage of penetrating injury compared to the total number of trauma injuries registered. The percentage of penetrating injuries increased from 6.7% in 2013 to 9% in 2019.

**Figure 4:**
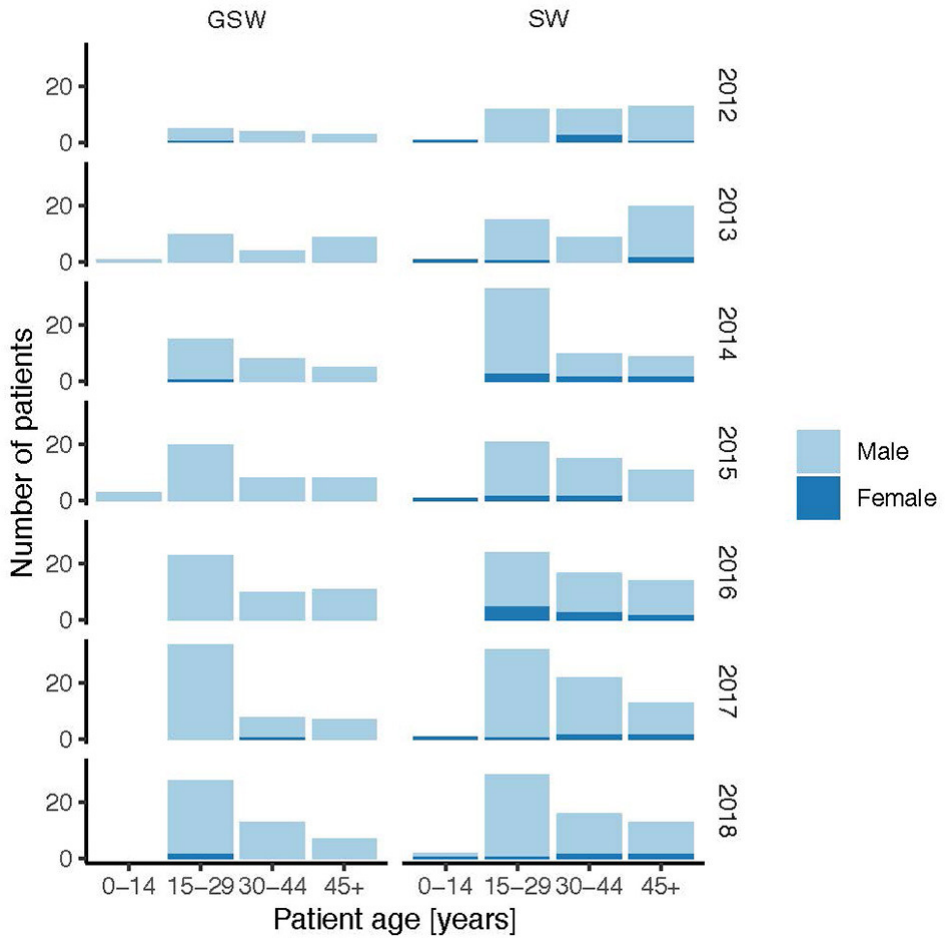
Age and sex distributions of the GSW and stab wound subgroups per year. GSW, gunshot wounds.

The age distributions of the GSW and SW subgroups per year are shown in Figure 4. Both for GSW and SW, the highest incidence was between 15 and 29 years, and the increase over the years involved mainly this age group. Very few cases of GSW have involved female patients over the years and were highest in 2018. However, the number was higher for SW and increased over the years.

**Figure 5:**
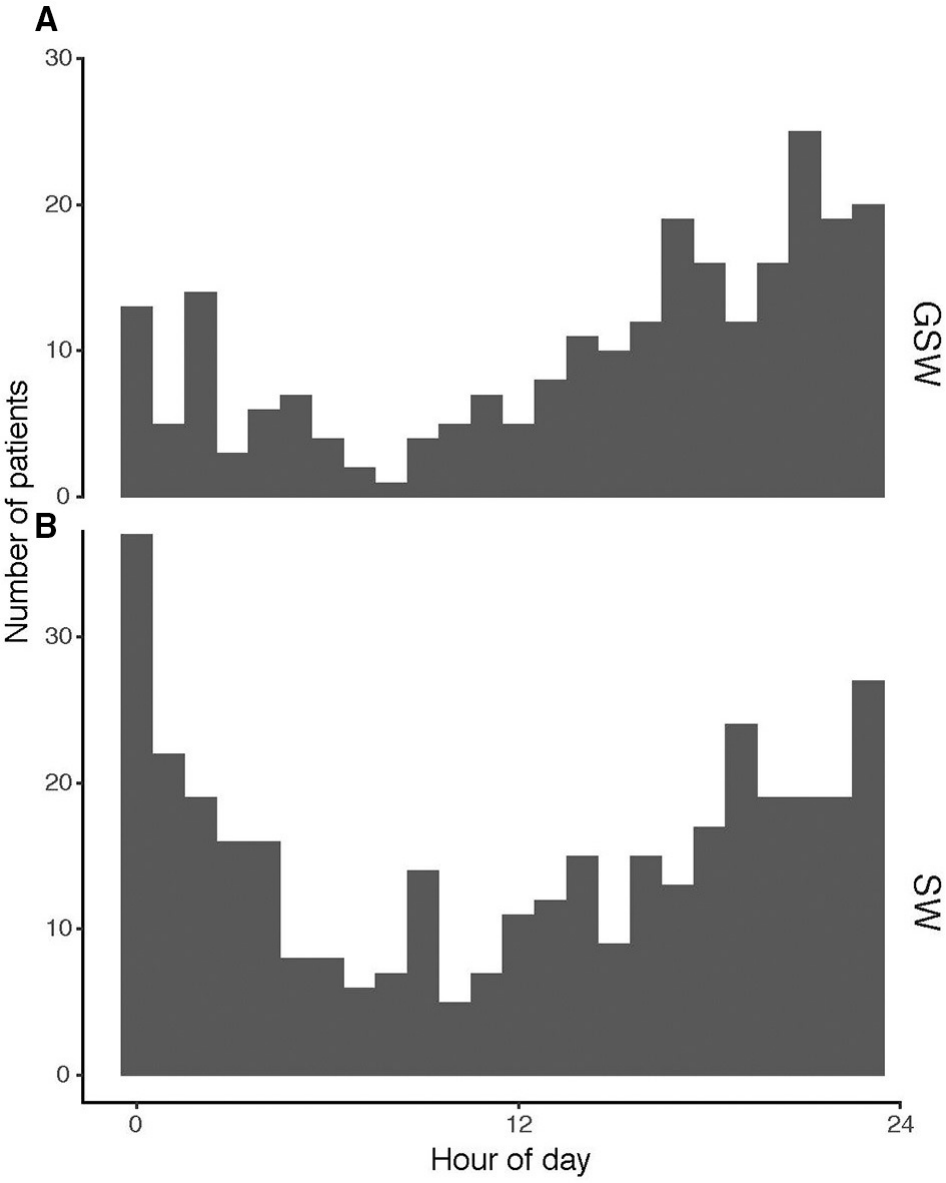
Time of day distributions for trauma in **(A)** GSW and **(B)** stab. GSW, gunshot wounds.

The time distribution of the GSW and SW subgroups per year is shown in Figure 5. The highest incidence of GSW occurred at 22:00 and the lowest incidence at 09:00. For SW, the highest incidence occurred at 00:00 and the lowest incidence at 09:00.

**Figure 6:**
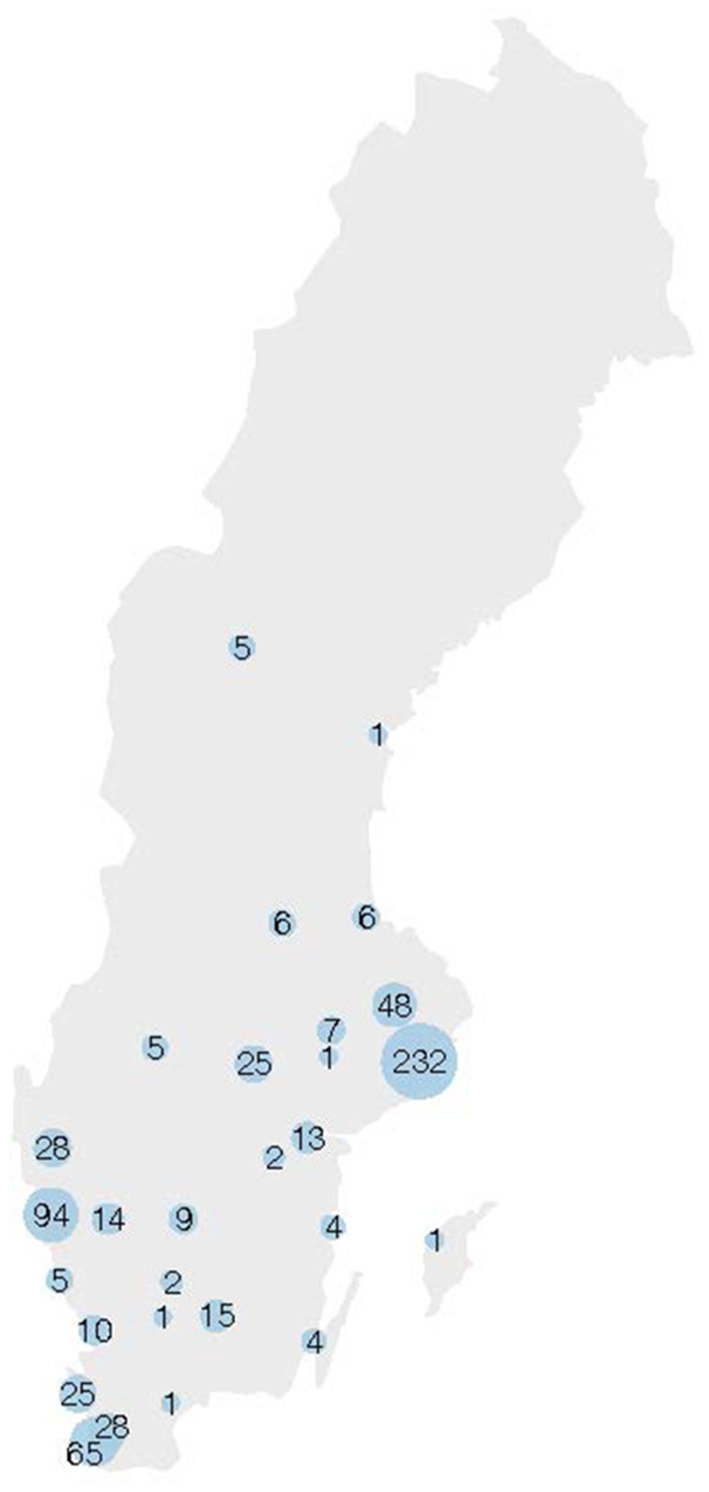
Cases of reported penetrating trauma with ISS ≥ 15 are plotted on a map of Sweden. The size of the blue dots correlates to the number of cases. ISS, Injury Severity Score.

The cases of reported penetrating trauma with ISS ≥15 were plotted on a map of Sweden (Figure 6), showing high levels of reported cases in the largest urban areas in Sweden: Stockholm, Gothenburg, and Malmö, while no traumas were reported from northern Sweden.

## Discussion

In this study, we report the largest, nationwide epidemiological study of penetrating injuries in Sweden, covering 7 years following the establishment of the SweTrau registry. Sweden has had a steady decrease in lethal violence from the early 1990s, until 2012, when there was a shift and the numbers started to rise. The increase in lethal violence has been linked to gang-related homicides with firearms (16). While the knife is the most common means of lethal violence, the use of firearms has increased steadily since the mid-00s and is today almost as common as knives (17). Interestingly, the majority of patients suffered injuries to the head, neck, and face and were associated with worse outcomes. One of our main objectives was to investigate whether a similar trend could be detected in the number of trauma admissions in hospitals. Our analysis from the SweTrau registry shows a steady increase in the number of SW and GSW since 2012. However, there is also a parallel increase in the coverage of the SweTrau registry that could affect these numbers. Although we cannot conclude that the actual numbers are precise for this time period, our comparison with Brå confirms an increasing trend in both SW and GSW.

We further assessed the 30-day in-hospital mortality. This was calculated as the fraction of the admitted cases and may, therefore, be less sensitive to register coverage. SweTrau only registers mortality in patients who had some kind of treatment initiated upon admission to a hospital. Interestingly, with the exclusion of 2012, there was no increase in 30-day mortality in GSW or SW. During the same time period, Brå reported an increase in violent deaths. Violence in Sweden became more deadly outside of hospitals, while in-hospital mortality was not affected. This may either reflect an increase in lethal violence on the streets, effective healthcare on the admission of the patients, or a combination of these.

As expected and previously reported, mortality was higher in patients with head trauma (4, 18). Patients with head trauma also had worse outcomes with only a minority in the upper scale of GOS. Although, surgical interventions were less frequent in this group which in combination with high mortality could indicate a more severe injury already at admission. However, further studies are needed to investigate contributing factors, such as the level of the trauma centers where these patients were admitted, interventions, and access to neurosurgeons and neuro-intensive care.

No robust trends of affected body regions could be detected, and the head-face-thorax-abdomen regions remained the most affected during the study period, which suggests a trend of more deadly violence on the streets, and that victims receive mortal penetrating injuries before reaching the hospitals. This may also be the reason why the number of cardiac arrests on admission remained low, and systolic blood pressures were clinically acceptable (around 120 mm Hg) on admission, despite severe injuries categorized as ISS ≥ 15.

A similar situation of increasing violence was reported from other European countries. SW was the most common type of injury (73%) followed by GSW (19%) in adult persons hospitalized for penetrating trauma injury in England and Wales between 2000 and 2005, with an increasing trend (9, 19). In Frankfurt am Main, Germany, out of 121 patients with penetrating injuries were caused by interpersonal violence or attempted suicide, 98 (81%) were caused by stabbing, and 23 (19%) were caused by firearms between 2008 and 2013 (8). Finland reported 130 penetrating traumas registered at trauma hospitals between 1997 and 2011 of which 16 (12%) were caused by a gun (20). In contrast, the US has an annual rate of hospitalizations for GSW of 80 per 1,00,000 hospital admissions, shown over 10 years (2004–2013) (21). A systematic review of penetrating injuries in the UK revealed an extensive range of incidence and mortality rates between studies in all regions, indicating the need for further studies directed toward the epidemiology of penetrating injuries within regional trauma networks (22). Sweden's incidence of GSW has become much higher than that of the comparable European countries mentioned above. The Swedish healthcare system has limited experience with these types of injuries, which is why further studies of the characteristics and management are needed, especially with trends of increasing violence. The severity of the injuries may also be reflected in the number of procedures performed on admission. Total 73% of trauma patients received a procedure on admission. In comparison, an urban major trauma center in the US reported that 75% of patients treated for GSW needed at least one surgical procedure during hospitalization (23). In Frankfurt am Main, 82.5% of penetrating trauma patients had surgical procedures performed (8).

Both GSW and SW had the highest incidence in 20–25 years of age. Although GSW did not show any change in age distribution during the years 2012–2018, SW had a trend of a lower peak age in 2018. In comparison, the median age of patients admitted for penetrating trauma in England and Wales was 30 years in a study excluding patients under 18 years (19). The mean age was 30.6 (range 13–70) years in London (24), 38 years (range 5–72) in Finland (20), and 38.9 (± 14.1) years in Frankfurt am Main, Germany (8). Therefore, the higher rate of firearm-related homicide victimization among men of 15–29 years in Sweden compared to other western European countries shown in criminology studies (10, 25) was also detected in higher hospital admission in this age group. The median age of SW and GSW admissions was lower in Sweden compared to other European countries.

The time-of-day distribution of admissions was highest at 22:00 and lowest at 09:00 for GSW and highest at 00:00 and lowest at 09:00 for SW. Most GSW admissions occurred on Saturdays. Majority of SW admissions occurred during Saturday nights and early Sunday mornings (data not shown). Characterization of time of incidence may contribute to the prevention, preparedness, and consequence management in healthcare. A similar admission pattern was reported from Frankfurt am Main, Germany, where a disproportionally high rate of penetrating trauma victims was admitted on Saturdays and Sundays (37.6%), and 63.4% of the patients with penetrating trauma were admitted between 16:00 and 08:00 (8). The injury localizations for GSW and SW were distributed in all regions of the body. A similar widespread injury pattern where mainly the head, thorax, and abdomen were affected was reported in Frankfurt am Main, Germany (8). Few other studies specify anatomic locations.

We report that 96.4% of lethal GSW injuries affected men. The Brå similarly reported that the gender ratio of male victims was an average of 90% in cases of lethal violence where a firearm was used, during the period 2011–2019 (11).

There are limitations in this study to be discussed. First, while currently 86% of Swedish trauma hospitals report to the trauma register, the number of centers reporting has increased during the study period. For this reason, we concluded that reliable incidence trends of admissions could not be assessed and consequently added comparisons with Brå. The 30-day mortality was defined as the percentage of admissions that may be less affected by coverage and the number of reported cases to the registry. Second, demographics and patient data, such as injury locations, were only reported for the patients admitted to hospitals, as only they were entered in the SweTrau register.

## Conclusions

Current results indicate an increase in penetrating injuries in Sweden which is also in line with Brå statistics. However, the 30 days mortality did not show a similar trend which could indicate more deadly violence on the streets and that victims receive deadly penetrating injuries before reaching the hospitals. The majority of patients had head trauma, which was associated with higher mortality and worse outcomes. Further studies are needed to investigate the role of possible contributing factors to worse outcomes in Sweden, such as the level of trauma centers, access to neurosurgery, and neuro-intensive care in the hope of improving the outcomes for these patients.

## Data Availability Statement

The data analyzed in this study is subject to the following licenses/restrictions: Application must be made to steering board of SweTrau. Requests to access these datasets should be directed to https://rcsyd.se/swetrau/.

## Ethics Statement

The studies involving human participants were reviewed and approved by Swedish Ethical authority (EPM). Written informed consent from the participants' legal guardian/next of kin was not required to participate in this study in accordance with the national legislation and the institutional requirements.

## Author Contributions

ER, AR, and FL conceived the idea of this study. ER, AR, MG, and MD made substantial contributions to the design of the study. MD and AA performed data curation. MD performed data analysis and statistics. All the authors agree to be accountable for all aspects of the study in ensuring that questions related to the accuracy or integrity of any part of the study are appropriately investigated and resolved and contributed to the interpretation of data, revised the manuscript critically for important intellectual content, and approved the version to be published.

## Funding

ER was a Wallenberg Clinical Fellow and funded by the Swedish Society for Medical Research. AR was funded by Myndigheten för samhällsskydd och beredskap (2019-13780).

## Conflict of Interest

The authors declare that the research was conducted in the absence of any commercial or financial relationships that could be construed as a potential conflict of interest.

## Publisher's Note

All claims expressed in this article are solely those of the authors and do not necessarily represent those of their affiliated organizations, or those of the publisher, the editors and the reviewers. Any product that may be evaluated in this article, or claim that may be made by its manufacturer, is not guaranteed or endorsed by the publisher.

## Abbreviations

SW: Stab wounds
GSW: Gunshot wounds
Brå: Swedish National Council for Crime Prevention
ISS: Injury severity score.
